# The Young Adult Centered Healthforce Training (YACHT) Program to Increase HIV Testing and Pre-Exposure Prophylaxis Referrals Among Young Sexual Minority Men in Florida: Protocol for a Type 2 Implementation-Effectiveness Hybrid Trial With a Stepped Wedge Design

**DOI:** 10.2196/63191

**Published:** 2024-11-20

**Authors:** Rebecca Giguere, Maria Isabel Fernandez, Jose A Bauermeister, Iván C Balán, Subhash Aryal, Andrea Cheshure, Sara Green, Willey Lin, Jonathan Morgan, Sylvie Naar

**Affiliations:** 1 Center for Translational Behavioral Science College of Medicine Florida State University Tallahassee, FL United States; 2 Department of Health College of Osteopathic Medicine Nova Southeastern University Miami, FL United States; 3 Department of Family and Community Health School of Nursing University of Pennsylvania Philadelphia, PA United States

**Keywords:** sexual minority men, youth, HIV prevention, tailored motivational interviewing, technical assistance

## Abstract

**Background:**

There is a high incidence of HIV among young sexual minority men in Florida. Many are unaware of their status due to low testing rates. Counseling, testing, and referral (CTR) services are essential for diagnosis and prevention of HIV and are integral to the Ending the HIV Epidemic (EHE) strategic plan. However, efforts to increase CTR among young sexual minority men have not been successful.

**Objective:**

The Young Adult Centered Healthforce Training (YACHT) program promotes developmentally sensitive, culturally appropriate, and evidence-based CTR services for young sexual minority men. This study tests whether the YACHT program increases HIV testing among young sexual minority men and fidelity to evidence-based CTR among testing providers.

**Methods:**

Agencies in Florida EHE counties that tested at least 24 young sexual minority men aged 18 to 29 years in 2021 will be invited to participate. The sites (N=42) will be randomized in blocks of 6 to participate in the YACHT program, following a stepped wedge design. Through YACHT, all sites will receive visits from mystery shoppers (MSs), who are trained to evaluate HIV testing services and complete postvisit quality monitoring assessments. Sites will be offered the opportunity to review their MS feedback and to receive tailored motivational interviewing training and evidence-based technical assistance to address areas of need identified through MS assessments. The study will evaluate whether YACHT leads to increased HIV testing by comparing numbers of young sexual minority men testing for HIV before versus after YACHT’s implementation. The Exploration, Preparation, Implementation and Sustainment framework will help understand the barriers to and facilitators of the program’s implementation and sustainment.

**Results:**

YACHT was funded in August 2022. Data collection began in June 2023. As of June 2024, 194 MS visits have taken place at 42 sites; 4 (67%) sites from the first block and 1 (33%) site from the second block have engaged with the study. At baseline, sites exhibited the lowest competencies in relationship context, counseling sessions, and safer sex education and the highest competency in privacy and confidentiality. Data collection will continue through May 2027, with results published by the end of 2027.

**Conclusions:**

To address the high incidence of HIV among young sexual minority men in Florida, YACHT aims to support testing sites with tailored motivational interviewing training and technical assistance to address needs identified by MS assessments. The program seeks to improve delivery of evidence-based CTR services, thereby increasing HIV testing, counseling, and pre-exposure prophylaxis referrals and reducing HIV incidence among this population.

**Trial Registration:**

ClinicalTrials.gov NCT06015581; https://classic.clinicaltrials.gov/ct2/show/NCT06015581

**International Registered Report Identifier (IRRID):**

DERR1-10.2196/63191

## Introduction

### Background

In Florida, as in many other areas of the United States, young sexual minority men, particularly those of color, bear a disproportionate burden of HIV infection [1,2]. HIV counseling, testing, and referral (CTR) services have long served as the gateway to prevention and care and are critical to reducing the impact of HIV among young sexual minority men. CTR services offers opportunities to address the “diagnose” and “prevent” pillars of the Ending the HIV Epidemic (EHE) plan through evidence-based practices (EBPs) such as risk reduction counseling (RRC), referral to pre-exposure prophylaxis (PrEP), early detection of HIV infection, and linkage to care. Despite concerted efforts at promoting uptake of HIV testing, only half of all young people living with HIV know their infection status [3]. Compared with other age groups, young sexual minority men are more likely to report never having undergone an HIV test [4] and are less likely to link and adhere to HIV treatment or biomedical prevention, including PrEP [5]. Reducing the devastating impact of HIV infection on young sexual minority men necessitates increasing access and uptake of CTR services among this heavily impacted population.

The HIV HealthForce is at the center of the EHE strategic plan and has been historically responsible for providing CTR services including HIV testing, discussing risk practices, encouraging risk reduction and repeat testing, identifying and addressing other psychosocial needs, linking to HIV care, and more recently, facilitating PrEP referrals. However, studies suggest that the HIV HealthForce is not adequately equipped to provide developmentally sensitive and culturally appropriate CTR services to young sexual minority men. In a recent assessment of CTR agencies by mystery shopper (MSs) to rate the services provided [6], young sexual minority men noted deficiencies in the testing providers’ competencies while working with sexual minorities, which led them to feel uncomfortable during their visits. Testing providers at about half of the CTR agencies visited by MSs missed opportunities for prevention by not providing EBPs such as counseling or PrEP referrals. Without developmentally and culturally responsive CTR services, outreach to increase testing uptake is less likely to be successful [7], young sexual minority men are unlikely to return for repeat tests [8], and the counseling and referral attempts are more likely to fail [9].

### Mystery Shopping Can Be an Effective Client-Centered Tool for Developing Targeted Workforce Development Plans to Improve CTR Services at Individual Sites

Mystery shopping [8,10] is an action-oriented strategy whereby organizations can understand their performance across a systematically obtained set of service indicators, as rated by the client base they intend to serve. MS data could be used to improve the quality of CTR services and establish a foundation from which to develop individualized workforce development (WD) plans that are tailored to the unique needs of each CTR site. Our team developed an MS assessment for CTR services driven by young sexual minority men and tested the measurement approach in 46 HIV and sexually transmitted infection (STI) testing clinics in the Detroit metropolitan area. We subsequently replicated the measurement approaches in Atlanta, Philadelphia, and Houston in Adolescent Trials Network (ATN) Protocol 139 [11].

For these studies, young sexual minority men from local communities served as MSs. They were trained to use a psychometrically sound quality assurance instrument to examine clinics’ environmental characteristics, compliance with federal and state EBPs, and delivery of developmentally and culturally competent CTR services. The MS sought services and completed the instrument immediately after completing the visit. A confidential 5-page report that documented site performance in comparison with other county agencies was provided to the agency directors. The directors expressed unanimous, positive support for how these reports helped advance their agency mission. However, beyond audit and feedback loops, quality management (QM) strategies must offer concrete WD strategies (eg, technical assistance [TA] and consultation) to help agencies address and monitor their improvement. Thus, an implementation package for the Young Adult Centered Healthforce Training (YACHT) program that includes MS and tailored motivational interviewing (TMI) WD strategies may be useful to improve CTR services for young sexual minority men in Florida.

### TMI Is a Promising Approach for Improving Testing Provider Competence in Delivering Evidence-Based CTR Services for Young Sexual Minority Men

There are several EBPs that should be standard as part of CTR services to young sexual minority men. These include counseling to encourage condom use and repeat testing, making referrals to PrEP services delivered from a motivational interviewing (MI) foundation to ensure collaboration, and providing nonjudgment and autonomy support to promote behavior change [12-19]. MI is a person-centered communication approach designed to elicit and strengthen motivation to change. This approach is particularly well suited for adolescents and young adults [20]. An additional benefit is that MI is flexible and thus, can be tailored to different contexts and geographic settings [7,21-25].

During the previous 2 decades, our team has conducted several studies to developmentally and culturally tailor MI with theory-driven training protocols resulting in an implementation package called TMI. The goal of TMI is to improve testing provider–patient communication across the youth HIV prevention and care cascades [7,21,26,27]. We have successfully used TMI in CTR settings [10]. The tailoring process was systematic and adolescent- and young adult–focused, and it used a variety of strategies. For the developmental tailoring, we analyzed recordings of real-world interactions with adolescents and young adults that happened during multidisciplinary clinic visits [7,23] and were guided by a seminal MI text focused on adolescents and young adults [20]. We culturally tailored MI through a series of communication science studies to specify testing provider behaviors most associated with the use of motivational language in ethnic minority populations [7,23,28]. To address stigma, we focused on incorporating more activities geared toward improving testing providers’ communication skills to demonstrate acceptance and support autonomy of patients [29,30]. There is evidence that increasing testing provider competence in these types of communication skills can reduce the manifestation of multiple and intersectional stigmas in the health care setting [31,32]. To further enrich TMI, we included activities to promote cultural humility [29]. Studies have shown that TMI can improve testing [7], linkage [33], retention [21], and viral suppression [21,27] in population samples comprised primarily of young sexual minority men of color.

However, delivering TMI with fidelity is not easy, and the MI literature suggests that workshops alone are not sufficient to improve testing provider competence; additional training activities are needed [34-37]. In our previous study of care providers for adolescents with HIV from different disciplines across 10 clinics in the United States (ATN 146), only 7% care providers scored in the intermediate or advanced TMI competence range, as assessed using a standardized assessment of simulated patient interactions [38]. This was despite the care providers reporting having received some prior MI training. To address this need, as part of our TMI implementation studies, we developed and tested a series of WD strategies to increase care provider competence in delivering TMI. These strategies include an introductory workshop, coaching and feedback sessions, standardized patient interactions, and performance-based coaching sessions [39]. Our studies suggest that these WD strategies were useful in enhancing care provider confidence and 100% adherence to all the strategies was not necessary [40].

Completing these WD strategies requires dedicated effort, and care providers in our studies cited time, funding, and competing demands as barriers to completing the strategies [40]. Innovative approaches, such as MSs, that identify improvement areas in the delivery of developmentally and culturally appropriate CTR services to young sexual minority men could be used to tailor the trainings and reduce the time burden. Linking MS methods with TMI WD strategies to enhance care provider–client communication could be a winning combination for improving CTR service delivery to young sexual minority men.

### Exploration-Preparation-Implementation-Sustainment Framework Offers a Rigorous Approach to Guide Implementation Strategies to Improve Health Force Fidelity to EBPs for Diagnosing and Preventing HIV Among Young Sexual Minority Men

Implementation science (IS) includes the study of strategies to adopt and integrate EBPs within specific settings to improve service quality and effectiveness. IS methods improve upon the historical approaches to studying structural interventions in several ways. IS provides theoretical frameworks to guide implementation strategy planning and to specify the assessment of barriers to and facilitators of success. The Exploration, Preparation, Implementation and Sustainment (EPIS) framework specifies 4 phases of the implementation process and delineates inner organizational and outer system variables that reflect the complex context in which interventions are implemented and scaled. The framework provides a reproducible mixed methods measurement approach. Our team has adapted the EPIS framework for IS studies in youth-focused HIV settings (ATN 153) [41,42]. Specifically, we developed an EPIS-based mixed methods framework for understanding youth HIV implementation contexts, and we have used this framework to organize implementation phases [37]. IS also offers new methods for rigorously testing such strategies, including hybrid trials and stepped wedge designs, which we leveraged in our previous studies. Finally, IS specifies QM and WD strategies to promote the uptake and fidelity of EBPs [43].

### Florida-Wide Initiative to Improve CTR Services to Young Sexual Minority Men Is Both Significant and Timely

With 7 EHE jurisdictions located in the state [44], Florida continues to battle a severe HIV crisis. For example, Miami, Orlando, and Jacksonville are consistently ranked among the top 10 US cities for new HIV diagnoses. Miami’s HIV incidence is 3 times more than that of the United States overall [1]. Although HIV rates are declining nationwide, 17 of Florida’s counties saw an increase in new diagnoses of HIV from 2018 to 2019, and approximately 76% were among racial and ethnic minorities (38% Black, 36% Hispanic and Latinx, and 2% American Indian, Asian, and mixed race) compared with 24% of the White population [45].

Florida has a large and ethnically diverse population, with almost half the population being people of color. It is also a state marked by great urban and rural contrasts and large disparities in wealth, health, age, and opportunities. Furthermore, the Florida Department of Health (FDOH) has an established and well-functioning HIV surveillance system and a strong track-record of public-academic partnerships.

### YACHT Implementation Package May Improve CTR Services to Young Sexual Minority Men

The YACHT implementation package follows the EPIS model to deliver MS feedback reports, TA in problem-solving identified areas of need, and TMI training to improve cultural humility and developmental appropriateness of HIV-testing services. We have partnered with FDOH to launch a state-wide initiative to test the YACHT implementation package. Through this collaboration, we are approved to access state surveillance databases as sources for pragmatic outcomes (number of HIV tests conducted among young sexual minority men). This state-wide testing of the YACHT implementation package paves the way for wide-scale sustainability and public health impact that can be documented with state-level HIV surveillance. This initiative could be a model for other state-wide implementation projects.

### Goal

In this manuscript, we present the protocol to test the effectiveness of the YACHT implementation package to improve the fidelity to the “diagnose” and “prevent” EBPs in CTR sites in Florida’s 7 EHE jurisdictions (Multimedia Appendix 1). Guided by the EPIS framework, we will examine both effectiveness (number of HIV tests conducted among young sexual minority men) and implementation (EBP fidelity) outcomes. After intervention implementation, a second randomization will be conducted to test whether the implementation of ongoing QM strategies (MS feedback reports) serves as a sustainment strategy. Our study also contains a qualitative component based on the EPIS framework to further understand the context of implementation and sustainment (sequential explanatory mixed methods) [46].

## Methods

### Study Design

**Figure 1:**
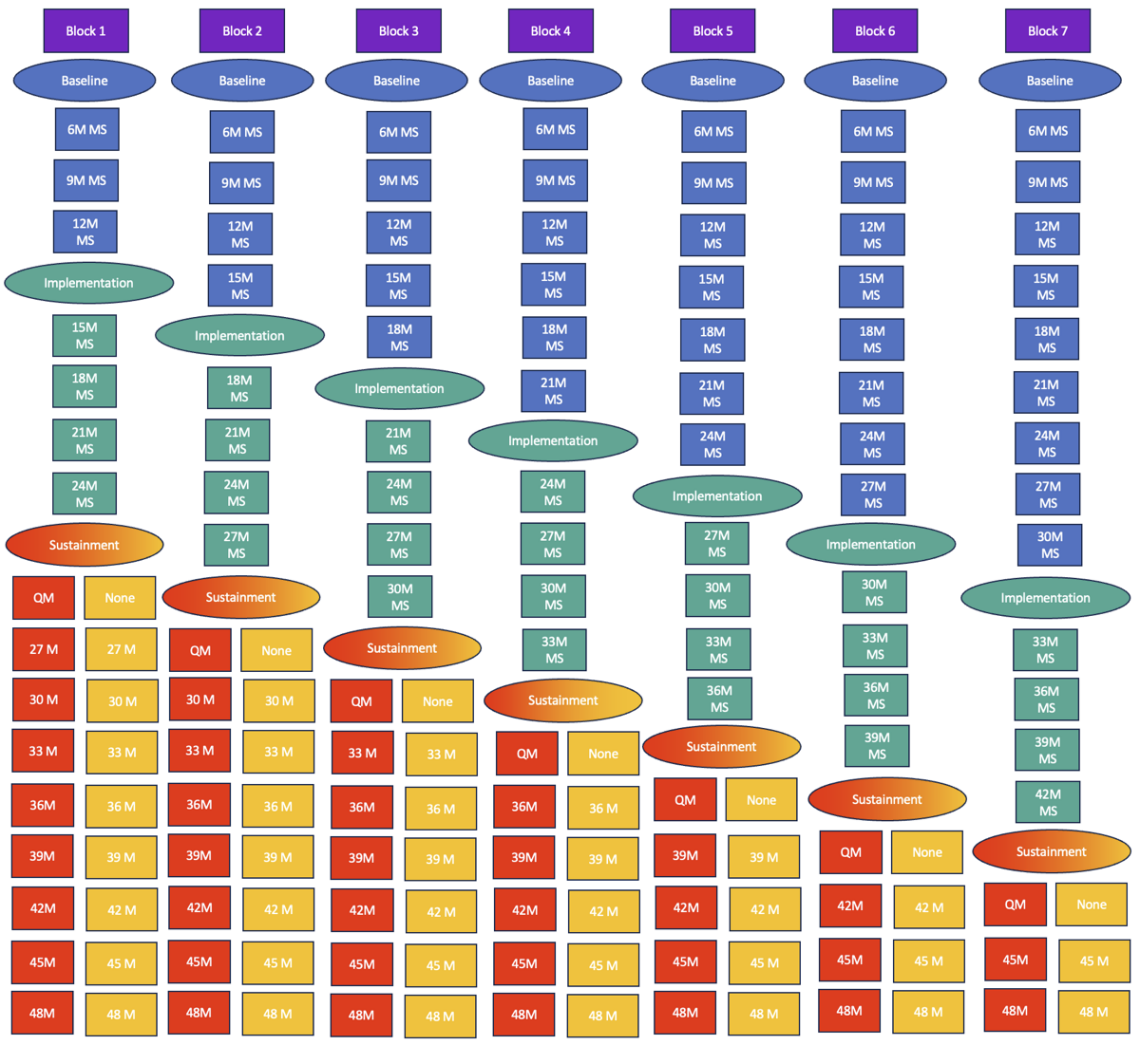
Stepped wedge study design. M: month; MS: mystery shopper; QM: quality management.

The YACHT program will be tested using a type 2 implementation-effectiveness hybrid trial with a stepped wedge cluster randomization (Figure 1). The trial comprises variable preimplementation phases (quarterly MS visits), a 12-month implementation phase (MS feedback along with TMI WD strategies and recommendations for improvement), and variable sustainment phase. For the stepped wedge, agencies will be randomly assigned to blocks. The number of assessments will be consistent across blocks, but baseline and sustainment windows will vary by block. The preimplementation phase will provide baseline data over a specified period before the implementation package is delivered. The preimplementation phase for block 1 will last for 12 months, whereas the preimplementation phase for block 7 (last block) will last for 30 months. Subsequently, each block will transition from preimplementation to implementation delivery at a 3-month interval. The implementation phase will last for 12 months and will be delivered to all the agencies within each block. MS visits will continue quarterly to inform implementation. At the end of the 12-month implementation period, the agencies will transition to the sustainment phase. Agencies within each block will be rerandomized to groups marked for either ongoing QM (MS feedback) or no further feedback (observation only). The length of the sustainment phase for block 1 will be of 24 months; for block 7, the sustainment phase will be of 6 months. We will conduct qualitative interviews with clinics’ testing providers and staff after the completion of the implementation and the sustainment phases using a sequential explanatory mixed methods approach. Implementation facilitators at each study site will collect written informed consent from the testing providers and staff before the interviews. Our logic model will guide the interview process (Textbox 1). The study will evaluate whether the YACHT program leads to an increase in HIV testing by comparing the number of young sexual minority men who test for HIV postimplementation versus preimplementation (primary effectiveness outcome). In addition, we will evaluate program implementation based on MS assessments of fidelity to developmentally and culturally responsive evidence-based CTR practices (implementation outcomes).

**Figure 2:**
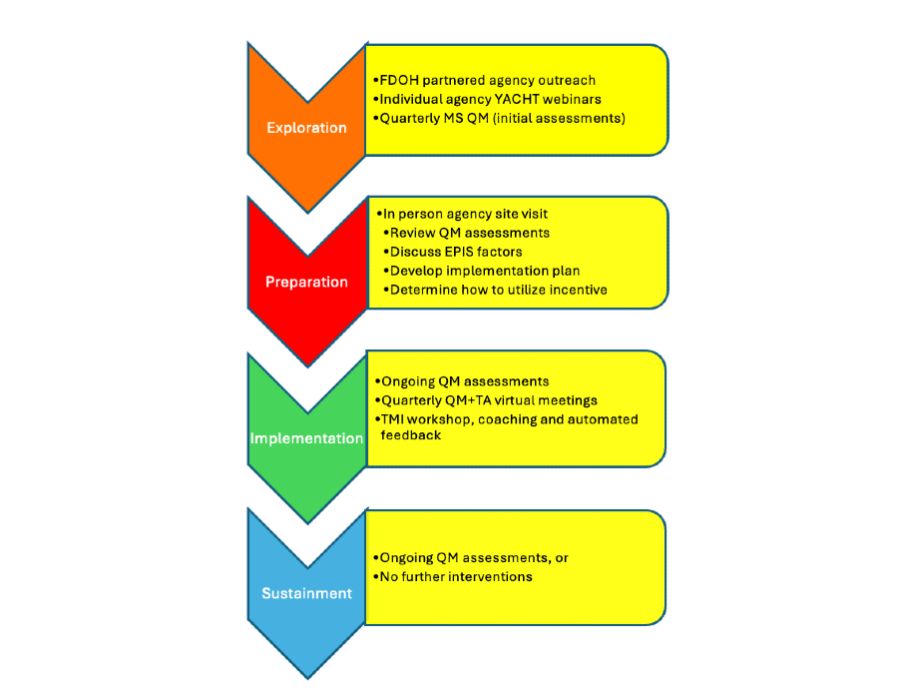
Young Adult Centered Healthforce Training (YACHT) Implementation Package by Exploration, Preparation, Implementation and Sustainment (EPIS) framework. FDOH: Florida Department of Health; MS: mystery shopper; QM: quality management; TA: technical assistance.

The study will use a multicomponent implementation strategy based on the EPIS framework (Figure 2). MS assessments will be used for quality monitoring, and WD strategies will include TMI training and evidence-based TA at HIV-testing sites. All testing sites in Florida EHE counties that tested at least 24 young sexual minority men aged between 18 and 29 years in 2021 will be invited to participate. Participating sites (N=42) will be randomized to receive the YACHT program in 7 blocks of 6, following a stepped wedge design (Textbox 1). They will receive TMI training for RRC and PrEP referrals and evidence-based TA for their identified areas of need, based on MS feedback. Finally, we will use the EPIS framework to understand the barriers to and facilitators of program implementation and sustainment.

This study design will accommodate real-world constraints and provide strong evidence for evaluating the research aims. It is carefully configured to optimize statistical power and will afford numerous and increasingly well-known benefits, including 2 comparisons for assessing the effect of the implementation package (ie, including inter- and intracluster comparisons for assessing the effect of the implementation package), evaluation and control of outer context factors, substantial rigor and power relative to quasi-experimental alternatives, and superior feasibility and power relative to alternative randomized designs. Institutional review board approval from the Florida State University was obtained on December 12, 2022.

**Logic model.**

**Determinants**

- Innovation
  Mystery shopper (MS) assessments and feedback shared with HIV testing sites
  Technical assistance and tailored motivational interviewing (TMI) offered to HIV testing sites
- Outer setting
  Ending the HIV Epidemic national initiative
  High HIV incidence among young sexual minority men in the state of Florida
  Florida Department of Health support for program
  Accountability and performance tied to funding
- Inner setting
  HIV testing already occurring at sites
  Culture at sites supports client centeredness in HIV testing and counseling
  Leadership is committed to staff development and data-driven practices
  Site incentives provide resources to support implementing the innovation
- Individuals
  High-level leaders, including research and clinical operations managers, engaged in exploration to identify need
  Mid-level leaders, such as HIV testing directors, engaged in preparation to discuss capability
  Implementation team members, such as HIV testing staff, engaged in implementation to support opportunity and motivation
- Process
  MSs will identify potential needs at each site
  Implementation facilitators (IFs) will share the needs assessment and collect information about priorities, preferences and context at testing sites
  IFs will engage mid-level leaders to plan strategies for implementation of innovation and adapt as needed
  Implementation team will together to carry out steps identified during the preparation phase
  IFs will assess barriers and facilitators to implementation to inform adaptation

**Strategies**

- Develop and implement tools for quality monitoring (MS)
- Audit and provide feedback
- Organize implementation team meetings: assess for readiness and identify barriers and facilitators
- Conduct ongoing training (TMI)
- Provide local technical assistance
- Provide ongoing consultation
- Provide financial incentives

**Mechanisms**

- Training testing providers/staff
  Increase cultural competence when working with young sexual minority men
  Promote skills in having sex-positive and inclusive conversations with young sexual minority men
  Increase knowledge of pre-exposure prophylaxis (PrEP) guidelines and referrals
  Strengthen motivation to discuss, refer, and link to PrEP
  Reduce stigma and promote equity during testing encounter
- Improvement of clinic procedures
  Destigmatize client environment
  Increase buy-in from staff on the value of PrEP
  Ensure adequate resources are available for clients who are young sexual minority men
  Take ownership of PrEP as a community response
  Create feedback loops to support accountability

**Outcomes**

- Implementation: improved MS scores in LGBTQ visibility, medical form inclusivity, clinic environment, privacy and confidentiality, PrEP information and dialogue, relationship context, participant counseling, safer sex education, perceived testing provider competency, testing provider interactions, cultural humility, and client centeredness
- Clinical/patient: increase number of young sexual minority men tested for HIV; increase number of young sexual minority men with PrEP referrals

### Ethical Considerations

This protocol was reviewed and approved by the institutional review board at the Florida State University on December 12, 2022 (study ID STUDY00003373). All participants who will be interviewed for this study will review and sign an informed consent form.

All study data will be deidentified. Reports of MS findings regarding individual study sites will only be reported aggregately outside of sharing the reports with site staff for quality monitoring purposes.

Study sites that opt to develop an implementation plan and collaborate with the study facilitator for ongoing quality monitoring and TA will be offered a US $3000 token of appreciation for their participation in the study. Study staff who complete a 4-session tailored MI training will receive continuing education credits. Finally, participants who are interviewed for the study will receive a US $25 gift card per interview, with a maximum compensation of US $50.

### Eligibility

Consistent with the partnering requirements of the funding opportunity announcement, we will be targeting all FDOH-contracted or direct service agencies (N=42) across all 7 EHE counties of Florida that meet the following eligibility criteria: (1) provide HIV CTR services and (2) have tested at least 24 young sexual minority men in 2021. FDOH has agreed to the participation of these 42 agencies. Given that a few agencies may cease operations or may be either unable or unwilling to adopt the implementation strategies, we conservatively estimate that we will need 36 agencies that have tested at least 24 young sexual minority men within each cluster.

### Hiring and Training of MSs

We will hire and train 4 to 6 MSs per region (north Florida, central Florida, and south Florida) to conduct QM assessments throughout the study period. We will use both active (eg, directly approaching young sexual minority men) and passive strategies (eg, posting advertisements on social media) to identify HIV-negative young sexual minority men (aged 18-29 years) who are interested in serving as MSs. We will also promote the opportunity via advertisements on various platforms. These platforms include lesbian, gay, bisexual, and transgender (LGBT) listservs; college listservs; HIV and AIDS community-based organizations; and local coffee shops and bars. In addition, we will use social media platforms and dating apps such as Jack’d (Perry Street Software), Facebook (Meta Software), Instagram (Meta Software), LinkedIn (Microsoft), and Idealist (Free Software Foundation).

MSs will participate in a training session that will follow the procedures we used in our previous studies [11]. Specifically, MSs will receive a structured 2-hour training session on the fundamentals of HIV or STI transmission, the guidelines and protocols surrounding HIV or STI testing and PrEP eligibility, and state-specific HIV guidelines and policies. MSs will also be trained to complete the assessment tool to optimize standardized data collection across agencies.

We will underscore the importance of being well versed in their rights and procedures, and increase skills on how to respond to worst-case scenarios (eg, how to turn down any unwanted procedures), were they ever to occur. MSs will be instructed to be honest about their sexual behaviors during their visits. For an HIV-positive test result, MSs will alert the study staff who will provide referrals for care, if needed.

MSs will be randomly assigned to each study site to avoid selection bias. The team will create a calendar for MSs to know their schedule of clinic visits. MSs will be instructed to report that they do not have income or health insurance and do not possess any proof of identification. By doing so, we will ascertain whether these are potential barriers to testing at a given location and document the lowest possible fees that may be charged to a young sexual minority man.

### MS Site Assessment Measure

MSs will complete a web-based agency checklist and qualitative debrief with study staff to summarize their impressions of the following: (1) how they felt about the agency, (2) interaction with the counselors and testing providers, (3) anything notable that occurred in the course of the visit (be it positive or negative), and (4) any other information deemed pertinent to the experience. Assessment training will include a discussion on the definition, understanding, and measurement of each mystery shopping domain (Table 1). The 11 mystery shopping domains include 9 that were previously used to assess HIV-testing sites in our previous studies along with 2 new domains focused on cultural humility and MI based on validated scales [47,48].

**Table 1:** Mystery shopping site assessment domains.

Domains	Items assessed	Interpersonal or environmental factor
Medical form inclusivity	Clinic used LGBT^a^-inclusive language on forms	Environmental
Clinic environment	Friendliness, sensitivity, use of affirming language by staff, lack of judgment, and comfort of waiting room	Interpersonal and environmental
Privacy and confidentiality	Patient info kept confidential by office staff and testing provider, privacy in waiting room, and confidentiality explained	Interpersonal and environmental
PrEP^b^ information and dialogue	Presence of PrEP information and whether testing provider shared info about PrEP as a prevention strategy	Interpersonal and environmental
Relationship context	Testing provider asked about sexual orientation, relationship status, sexual activity, and experiences with intimate partner violence	Interpersonal and environmental
RRC^c^	Testing provider explored motivation for testing, offered to help set safer sex goals and action steps, and presented risk reduction options	Interpersonal
Safer sex education	Testing provider helped me to identify condom and lube that work for me and made sure I know how to use them, and discussed PrEP as prevention strategy	Interpersonal and environmental
Testing provider knowledge	Testing provider was knowledgeable about HIV and STIs^d^ and LGBT health issues	Interpersonal and environmental
Testing provider interactions	Testing provider made me feel comfortable, was not judgmental about my sexual activity or partners, did not pressure me to adopt risk reduction options	Interpersonal
Cultural humility	Testing provider was knowledgeable, understanding, or curious about my cultural background, or made assumptions about my cultural experiences	Interpersonal
Client-centeredness	Clinic staff took a collaborative stance when discussing making healthy changes, supported my autonomy, helped me recognize need for change and feel confident in my ability without acting as an authority	Interpersonal

^a^LGBT: lesbian, gay, bisexual, and transgender.

^b^PrEP: pre-exposure prophylaxis.

^c^RRC: risk reduction counseling.

^d^STI: sexually transmitted infection.

### YACHT Implementation Package Specification

#### Overview

During the exploration phase, we will present at state-wide meetings and conferences to inform agencies state-wide about the initiative in collaboration with FDOH. Every quarter, each study site will receive 2 MS visits. We will use time-space sampling to ensure that varying days (weekdays vs weekends, when applicable) and times (eg, morning vs afternoon or evening) are selected for visits, thereby maximizing the chance of meeting different counselors at each agency. The exploration phase will end with virtual meetings of individual agencies with regional facilitators to orient HIV-testing directors to the program, address any initial concerns, and increase engagement with the planned implementation strategies. These meetings will aim to create an engaging, interactive environment to probe knowledge and utility of MSs for HIV-testing programs. Regional facilitators will describe the MS assessment and approaches using TMI and TA, ending the session with an overview of the study and scheduling an in-person visit for the preparation phase.

The preparation phase will occur 2 months before the implementation phase and will consist of an in-person meeting with the executive director and the leads of testing and prevention services. We will provide personalized feedback using the MS assessment findings that address environmental and interpersonal factors. We will elicit their views regarding the findings, focusing on the aspects they would like to improve. We will also suggest strategies for change and assist them with planning an intervention appropriate for their agency. To address environmental factors, we will lead agency directors in a problem-solving exercise to brainstorm ideas and develop a plan to address the components they select. To address the interpersonal factors, we will discuss how TMI may be used to improve testing provider–client interactions and discuss challenges and opportunities for improving CTR services. In collaboration with the agency, we will compile a list of staff to engage in the intervention. Consequently, the YACHT program will be adapted to each agency’s needs and culture.

The implementation phase (12 months) will consist of quarterly 1-hour QM meetings in which we will review data regarding adherence to the implementation plan (eg, staff attendance at TMI training) as well as the most recent MS assessments, iteratively revising the implementation plan as needed. TA tailored to the areas of need identified during the preparation phase will be offered throughout the implementation period. We will use a facilitated problem-solving approach consisting of the following 4 steps: (1) define and analyze the problem, (2) generate new ideas, (3) analyze potential solutions, and (4) implement and evaluate the plan. In addition, to build testing provider’s interpersonal counseling skills, we will offer TMI options as follows:

#### Initial TMI Workshop

This workshop is theoretically grounded in behavior modification and behavioral skills training developed by Bandura [49] and Miltenberger [50] as well as research in education on cooperative learning environments [51,52]. Originally designed as a 2-day in-person training, we will deliver TMI workshop virtually via structured web-based modules followed by two 3-hour interactive modules on Zoom with cooperative learning activities, video examples, and behavioral skills acquisition steps (modeling, verbal and behavioral rehearsal, and feedback). We have further adapted these TMI modules to ensure delivery from a foundation of cultural humility [53] and antiracism [54], emphasizing how learners engage in a lifelong process of self-reflection and self-critique, challenge power dynamics, commit to understanding and respecting different points of view, and engage with others authentically and from a place of learning. Discussion sessions will focus on moving from not racist to antiracist while concepts of cultural humility with video examples will be interwoven throughout training. Participants will practice how to use TMI skills to demonstrate cultural humility in language with video demonstrations.

#### Coded Standard Patient Interactions With Automated Feedback

After participating in the workshop, testing providers will complete a 15- to 20-minute long standard patient interaction by phone. TMI coaches will code the interactions with the Motivational Interviewing Coach Rating Scale (MI-CRS) [47,55]. The MI-CRS consists of 12 items rated on a 4-point scale (beginner, novice, intermediate, and advanced) representing essential MI components such as a collaborative stance, autonomy support, open questions to elicit motivational language (ie, change talk), reflections of change talk, affirmations, cultural humility, and summaries. Testing providers will receive an autogenerated report based on scores with recommendations for practice activities and links to short videos of skill demonstration, including newly developed cultural humility videos, based on the lowest scoring items. These scores will guide the group coaching (discussed next) and will also be repeated at 6 months to provide further feedback.

#### Group Coaching

Standardized 1-hour virtual coaching sessions (up to 4 per agency) will include a brief interaction to elicit motivation for TMI implementation and then, behavioral skills acquisition based on the lowest ratings received on the most recent MI-CRS and MS assessments. The coaching of each skill will follow the skills acquisition steps of modeling, verbal and behavioral rehearsal, and feedback.

In the sustainment phase, agencies will be randomized to either continue receiving written feedback from the quarterly MS assessments or no additional feedback. Agencies will not have any contact with the TMI coaches during this period; however, agencies may continue to seek TA from FDOH as standard of care.

#### EPIS Fidelity Procedures

In the exploration phase, MSs will complete the site assessments. We will also have site communication logs in which the facilitator for each study site will document contact with sites, including e-mails, phone calls, and meeting notes. In the preparation phase, each site will have a personalized feedback report based on the MS assessments, and we will continue to document contact in the site communication logs. We will also have a checklist of completed steps and of potential facilitators of and barriers to engagement in implementation. Finally, we will develop and document an implementation plan for each site. In the implementation phase, we will document (1) review of new MS data and any agency data regarding testing, (2) review of adherence to implementation plan, (3) provision of TA to overcome barriers and enable facilitators, (4) revision of implementation plan, and (5) development of sustainment plan at the last QM meeting. Furthermore, 1 TMI group coaching session per block will be randomly selected and coded by the study’s principal investigator for content and TMI consistency with the MI-CRS. Finally, agency directors, supervisors, and staff will be interviewed to determine barriers to and facilitators of YACHT fidelity. In the sustainment phase, agencies will be referred to health departments for ongoing TA and another round of interviews will be conducted at the end of the phase with the same staff to identify the barriers to and facilitators of sustaining EBPs.

#### Quantitative Measures and Data Analysis

Data are nested, which will be addressed using mixed-effects regression models. The key feature is that a minimum of 36 (out of 42 recruited) agencies are nested within the 7 counties. We will explore the use of random intercept at the county level. With only 7 counties, our model may run into convergence and estimation issue. In such a situation, we will include appropriate fixed-effects indicators in our model. We will request FDOH data on the number of HIV tests administered in quarterly intervals during all 3 phases (preintervention, intervention, and sustainment).

#### Primary Effectiveness Outcome

The primary outcome is the number of tests completed by young sexual minority men (aged 18-29 years), as reported in aggregate at the agency and county levels. We expect that the YACHT package will improve the number of HIV tests administered to young sexual minority men at each agency. As an exploratory outcome, FDOH has recently begun tracking PrEP uptake. Both the testing and PrEP data are reported in a standardized fashion to FDOH. Data will be aggregated within agencies and will be requested biannually. We have budgeted for staff at FDOH to manage data and deliver it in analyzable format. Data will be managed into quarterly windows to correspond to the data collection intervals in the stepped wedge design.

#### Analytic Strategy

We will use descriptive and graphical approaches to describe demographic characteristics, evaluate distributional properties, and identify outliers. The primary outcome of this study is the number or rate of HIV tests among young sexual minority men at each of the 42 FDOH-contracted CTR agencies in the 7 Florida EHE counties. The secondary outcome of this study is the mean score changes across MS domains at each of the FDOH-contracted CTR agencies. We will use generalized linear mixed models to evaluate our outcomes under the intention-to-treat analysis framework. Specifically, we will explore the use of the mixed-effects negative binomial model or the mixed-effects Poisson regression model for estimating risk ratio between the treatment and control arms for the testing data, and a mixed-effects model with a normal distribution for the MS data. Time will be included in the model as continuous exposure. Our models will evaluate the difference between the intervention and control arms, adjusting for calendar time and exposure time, which is recommended by Nickless et al [56] to reduce bias in stepped wedge cluster randomized trials. To account for correlation between multiple tests conducted within each agency and heterogeneity between agencies, we will include an agency-specific random effect term. We will also explore the inclusion of a higher order quadratic term for time for better model fit. Akaike information criteria and Bayesian information criteria will inform final model selection. We will calculate the intervention effect after 3, 6, 9, and 12 months of exposure and the time-averaged intervention effect. We will report 95% CI and corresponding *P* values for our parameter estimates. At the conclusion of the 1-year intervention period, agencies will be randomized to continuous quality improvement versus control groups to evaluate sustainment over time. The sustainment period will be 24 months for agencies that receive intervention during the first step and 6 months for agencies that receive intervention during the last step. We will again use mixed-effects regression models to evaluate the difference between continuous quality improvement versus control groups over time.

We will conduct sensitivity analysis to evaluate a per-protocol effect of the intervention on the number of HIV tests administered to young sexual minority men. We will extend our generalized linear mixed models framework to examine the presence of mediation and moderation effects in our models. Specifically, we will examine whether there is a moderating relationship between agencies using our implementation strategies (eg, TMI coaching attendance, number of TA sessions requested, prospective improvements in domains assessed by MSs) and changes in the number of HIV tests administered to young sexual minority men over time.

#### Attrition and Missing Data

The analyses will follow an intention-to-treat approach, with observations retained in the randomly allocated cluster and phase independently of participation in the experimental interventions. Highly successful tracking and retention protocols will minimize missing data. However, some data will inevitably be missing, and multiple methods will be used to evaluate assumptions and guide the analyses: (1) for a small proportion of missing data with evidence of missing at random, the data will be analyzed using the estimation procedures described above; (2) for a nontrivial amount of missing data and evidence of missing at random, multiple imputation for repeated measurements will be used to generate complete data; and (3) for a nonrandom missing data mechanism, pattern mixture models will be used to identify and control the effect of the nonrandom mechanism.

#### Primary Implementation Outcomes

Using the pre-established MS domains in Table 1, we will compute an overall agency score using the pooled scores from the 2 MS visits conducted each quarter. Pooled scores will be presented to reduce potential selection bias and confounding based on whether the same or a different testing provider interacted with the shoppers at either agency visit, and to account for the variability across shoppers. We will also create domain subscores using our psychometrically tested subscales for (1) LGBT visibility, (2) medical form inclusivity, (3) perceived clinic environment, (4) privacy and confidentiality, (5) relationship context, (6) RRC, (7) safer sex education, (8) PrEP information and referral, (9) perceived testing provider competency, and 10) patient–testing provider interactions. For ease of interpretability across domains, we will normalize the pooled average scores into percentiles.

#### Secondary Implementation Outcomes

##### Secondary Aim 1: To Assess the Effect of Continuous Quality Improvement (Ongoing MS Feedback) in Sustaining the Primary Outcomes

The data structure is identical to that described for the primary effectiveness outcome. To evaluate the outcomes in the sustainment period, including the number of tests administered to young sexual minority men and MS scores, a dichotomous indicator will be added at the agency level to differentiate agencies randomized to continued MS feedback from those randomized to no further intervention. In the model detailed for the primary effectiveness outcome, cross-level interactions will be specified between this condition indicator and the level-2 sustainment phase indicator, along with the level-1 growth term for the sustainment phase. This will test the extent to which changes in outcomes during the sustainment phase differ for agencies receiving ongoing MS feedback versus the control arm. Likewise, the model can be simplified to test for a difference in the average level of MS scores and young sexual minority men tested rather than change over time during this phase.

##### Secondary Aim 2: To Use Mixed Methods Based on the EPIS Framework to Understand Barriers to and Facilitators of Successful Implementation and Sustainment of Youth-Specific EBPs

Up to 5 testing providers per agency will be interviewed immediately after the Implementation and Sustainment periods using the EPIS-based HIV interviews developed in preliminary studies. Accordingly, the interviews (45-60 min) will focus on the inner and outer context factors influencing the implementation of RRC, PrEP referral, and TMI; utility of and adherence to the YACHT strategies; and bridging and innovation factors to consider for future scalability and sustainment. We will use rapid content analysis as recommended to produce actionable information for planners and decision makers [56,57] by first summarizing individual interviews using a rapid feedback template of EPIS constructs and then consolidating the summaries by the main EPIS factors. Next, we will adapt the innovation profile approach [58] originally developed for classroom research. This approach results in a multidimensional rubric to classify where an organization is in the process of developing its capacity to engage in a particular set of activities; in this case, it is the integration of EBPs into routine service provision. The dimensions and subdimensions of the matrix we develop, as well as descriptions of the behavioral indicators of exemplary, intermediate, emerging, and low capacity to integrate EBPs will be derived from aggregating the data produced during content analysis at the agency level. These data will be integrated with the quantitative fidelity and the outcomes data using a sequential explanatory design where qualitative findings are used to help explain variability in fidelity and outcomes [59].

## Results

The study was funded on August 4, 2022. A simple randomization scheme was generated using SAS (version 9.4; SAS Institute) to assign agencies to receive feedback versus no feedback during the sustainment phase on April 18, 2023. MS visits were initiated on June 1, 2023. As of June 2024, 194 MS visits have taken place at 42 sites. In addition, 4 sites from the first block and 1 site from the second block have engaged with the study. At baseline assessment, sites exhibited the lowest competencies in relationship context, counseling session, and safer sex education, and the highest competency in the privacy and confidentiality domain.

The exploration phase is projected to last from June 1, 2023, to March 31, 2024 (10 months), for block 1 and until September 30, 2025, for block 7 (28 months), with the other blocks staggered every 3 months. The preparation phase for block 1 started on April 1, 2024, and is projected to take up to 2 months for each block. The implementation phase will last for 1 year and started on June 1, 2024, for block 1 and will begin on December 1, 2025, for block 7, with other blocks staggered every 3 months. Finally, the sustainment phase will start on June 1, 2025, for block 1 and last for 2 years. For block 7, it will start on December 1, 2026, and continue for 6 months. Data collection will be completed by May 31, 2027.

## Discussion

### Principal Findings

We hypothesize that the YACHT implementation package will contribute to increasing the number of HIV tests conducted among young sexual minority men and improving the developmentally and culturally responsive delivery of EBPs by participating sites in Florida’s 7 EHE jurisdictions. An important innovation of our proposed approach is using the services of MSs who are young sexual minority men to identify areas of strength and improvement in the delivery of CTR services for young sexual minority men at participating sites. The proposed design guided by the EPIS framework, a type 2 implementation-effectiveness hybrid trial with a stepped wedge cluster randomization, will allow us to examine both effectiveness outcomes (number of HIV tests on young sexual minority men) and implementation outcomes (EBP fidelity). The second randomization conducted after intervention implementation will test whether the delivery of ongoing QM (MS feedback reports) serves as a sustainment strategy. The qualitative component will advance our understanding of the context of implementation and sustainment.

MS visits started on June 1, 2023. We have learned that recruitment of MSs is challenging and often requires more resources than originally allocated. Recruitment via dating apps such as Jack’d has been most successful but requires an advertising budget that is more extensive than simply posting on social media or posting flyers in local universities and cafes. Interest in serving as an MS varies by geographic location. Scheduling and MS retention can be demanding and often requires extensive efforts from research assistants. The current compensation structure for MSs merits further examination. Most MSs are neither university employees nor study participants but consultants. We pay them on a per visit basis as has been done in previous work with young sexual minority men [53]. However, timeliness of the payment per University processes prevents immediate payment upon visit completion, which may also deter engagement of MSs.

We have some preliminary evidence that garnering participation of some CTR sites may be problematic, even though we have the full support of the FDOH. In February 2023, directors of the CTR sites in the EHE jurisdictions were sent an opt out letter describing the study and giving them the opportunity to opt out of the MS visits. Although a small percent of sites opted out after the initial mailing, a few sites opted out after our follow-up calls and others did not return our calls. As a result, we have ramped-up our site engagement efforts by conducting outreach to both FDOH and county HIV and AIDS prevention coordinators to promote the study and to verify contact information for site directors. We are currently contacting sites in the first 3 blocks through an exploration engagement email, with plans to follow-up over phone if no response is received. To date, we have engaged 8 sites in exploration visits and 3 sites in preparation visits. By emphasizing our plan to share MS data and to provide site incentives (eg, continuing education units for staff that complete the full TMI training and a US $3000 incentive for each site that elects to participate and develop an implementation plan), we hope to continue to garner interest in the study as it is beneficial for the testing agencies.

### Limitations, Foreseeable Challenges, and Potential Solutions

Staff turnover is a threat to any health force training. All trainings will be recorded so that the recorded trainings along with our TMI video examples will allow new testing providers to join the program even if they miss the original workshop. In addition, TMI webinar trainings will be offered on a rolling basis for all agencies. In addition, each agency’s implementation plan will be clearly documented and we will ensure they have a copy they can refer to and share with new staff. Another potential challenge is that agencies may close or lose funding and drop out of the study. We have included all 42 eligible agencies to reduce bias, but power calculations suggest that the study may be completed with as few as 30 agencies. In addition, the political context in Florida is currently challenging for HIV-testing sites that target young sexual minority men because images and language that explicitly support LGBT youth are banned. As a result, the evaluation and discussion of developmental and cultural aspects of CTR for young sexual minority men must be tailored to be both acceptable and clear, without penalizing sites for political factors they cannot control. Finally, MSs are likely to turn over, but we have budgeted for additional outreach and training time for new shoppers.

### Conclusions

Improving the CTR service delivery to young sexual minority men has the potential to reduce the impact of HIV on this underserved, at-risk group of young people. Our study provides an innovative strategy for identifying areas of strengths and improvement and an implementation package to address these needs. If successful, the MS visits coupled with the YACHT implementation package will provide a scalable model to improve CTR services that could be adapted for other underserved populations.

## Appendix

Multimedia Appendix 1 Funded peer review.

## Abbreviations

ATN: Adolescent Trials Network
CTR: counseling, testing, and referral
EBP: evidence-based practice
EHE: Ending the HIV Epidemic
EPIS: Exploration, Preparation, Implementation and Sustainment
FDOH: Florida Department of Health
IS: implementation science
LGBT: lesbian, gay, bisexual, and transgender
MI: motivational interviewing
MI-CRS: Motivational Interviewing Coach Rating Scale
MS: mystery shopper
PrEP: pre-exposure prophylaxis
QM: quality management
RRC: risk reduction counseling
STI: sexually transmitted infection
TA: technical assistance
TMI: tailored motivational interviewing
WD: workforce development
YACHT: Young Adult Centered Healthforce Training

## Data Availability

Data sharing is not applicable to this paper as no datasets were generated or analyzed during this study.
