# Supplementary material for: The Young Adult Centered Healthforce Training (YACHT) Program to Increase HIV Testing and Pre-Exposure Prophylaxis Referrals Among Young Sexual Minority Men in Florida: Protocol for a Type 2 Implementation-Effectiveness Hybrid Trial With a Stepped Wedge Design
Source: JMIR Res Protoc. 2024 Nov 20;13:e63191. doi: 10.2196/63191 (PMC11618015; doi:10.2196/63191)
Supplement: Multimedia Appendix 1 [file resprot_v13i1e63191_app1.pdf]

SUMMARY STATEMENT

PROGRAM CONTACT: **(Privileged Communication)** ***Release Date:*** **11/30/2021**
Usha Sharma ***Revised Date:***
240-292-4809
usharma@niaid.nih.gov

Application Number: **1 R01 AI169980-01**
Principal Investigators (Listed Alphabetically): ***Formerly:*** **1R01MH130047-01**

BAUERMEISTER, JOSE ARTURO
FERNANDEZ, MARIA ISABEL
NAAR, SYLVIE (Contact)

Applicant Organization: FLORIDA STATE UNIVERSITY

Review Group: **ZAI1 DNV-A (J3)**
National Institute of Allergy and Infectious Diseases Special Emphasis Panel
Prevention Strategies to End the HIV Epidemic (R01 Clinical Trial Optional)
AIDS - EXP. REV.

Meeting Date: **11/18/2021** ***RFA/PA:*** **AI21-025**
Council: **JAN 2022** ***PCC:*** **A22A**
Requested Start: **03/01/2022** ***Dual PCC:*** **9A-ASG**
Dual IC(s): **DA, MH**

Project Title: **Scaling Up Implementation Strategies to Improve the DIAGNOSE and PREVENT Pillars for Young MSM in Florida**
SRG Action: **Impact Score:32**
Next Steps: **Visit https://grants.nih.gov/grants/next_steps.htm**
Human Subjects: **30-Human subjects involved - Certified, no SRG concerns**
Animal Subjects: **10-No live vertebrate animals involved for competing appl.**
Gender: **1A-Both genders, scientifically acceptable**
Minority: **1A-Minorities and non-minorities, scientifically acceptable**
Age: **3A-No children included, scientifically acceptable**

Project Year	Direct Costs Requested	Estimated Total Cost
1	533,258	814,095
2	690,813	1,054,626
3	758,340	1,157,715
4	685,079	1,045,872
5	636,830	972,213
TOTAL	3,304,320	5,044,521

ADMINISTRATIVE BUDGET NOTE: The budget shown is the requested budget and has not been adjusted to reflect any recommendations made by reviewers. If an award is planned, the costs will be calculated by Institute grants management staff based on the recommendations outlined below in the COMMITTEE BUDGET RECOMMENDATIONS section.

1R01AI169980-01 Naar, Sylvie

RESUME AND SUMMARY OF DISCUSSION: In this application entitled “Scaling Up Implementation Strategies to Improve the DIAGNOSE and PREVENT Pillars for Young MSM in Florida” the Principal Investigator proposes to implement strategies aimed at improving counseling, testing and referral services (CTR) for young men who have sex with men (YMSM) in the state of Florida. Studies by the PI and team using Mystery Shoppers underscored the lack of preparedness in the delivery of developmentally appropriate, culturally competent CTR services to YMSM. To this end, the PI and team propose to properly train and support CRT workforce in delivering improved services to YMSM while leveraging Mystery Shoppers in evaluating the intervention. The reviewers agree that the proposed studies are highly significant given they address a major barrier to HIV testing and PrEP referral in YMSM. The use of Mystery Shoppers as well as the inclusion on YMSM in assessing the outcomes of this intervention is seen as highly innovative. Other major strengths include the experienced investigative team, the feasibility of the proposed work, and the leveraging of the team’s prior work on the impact of tailored motivational interviewing in HIV testing and prevention. Minor weaknesses that did not impact an otherwise excellent application include the lack of clarity on the mystery shopper approach, the limited justification of the intervention phases, as well as how the intervention will impact structural factors. Nevertheless, the panel agreed that this is an excellent application from an experienced investigator that can have a major impact on enhancing CTR practices and ultimately HIV prevention.
Based upon the evaluation of scientific and technical merit, this application received an Impact/Priority score of 32.

DESCRIPTION (provided by applicant): This project will launch a Florida wide effort to promote the delivery of developmentally sensitive, culturally appropriate and evidence based DIAGNOSE and PREVENT counseling testing and referral services (CTR) for young men who have sex with men (YMSM). The goal is to leverage implementation science strategies to improve the capacity of the HIV HealthForce to deliver evidence-based practices (risk reduction counseling, PrEP referral and Tailored Motivational Interviewing) within CTR services. Our recent YMSM Mystery Shoppers studies conducted at CTR sites in three cities indicated that providers are woefully unprepared or do not know how to deliver developmentally appropriate, culturally competent CTR services to YMSM and miss opportunities to deliver these evidence-based practices. Mystery Shopper is a quality management strategy to monitor implementation fidelity to culturally and developmentally responsive EBPs in CTR settings. However, assessment and feedback alone are insufficient to improve fidelity; the HealthForce must be properly trained and given technical assistance. We propose integrating two implementation strategies: quality management Mystery Shoppers and HealthForce training in Tailored Motivational Interviewing with centralized technical assistance. We will test Young Adult Centered HealthForce Training (YACHT) package in Florida’s seven EHE counties among the Department of Health’s 42 contracted sites who delivered CTR to at least 24 young MSM in the previous 12 months using a Stepped Wedge design. We power on both effectiveness outcomes (# of tests of YMSM) and implementation outcomes (EBP fidelity based on Mystery Shopper Assessments) consistent with a Type 2 Hybrid trial.

PUBLIC HEALTH RELEVANCE: This study will significantly contribute to the field of HIV prevention and treatment by testing an intervention that helps counseling and testing agencies contracted by Florida Department of Health to improve their services to young men who have sex with men.

CRITIQUE: The comments in the CRITIQUE section were prepared by the reviewers assigned to this application and are provided without significant modification or editing by staff. They are included to indicate the range of comments made during the discussion and may not reflect the final outcome. The RESUME AND SUMMARY OF DISCUSSION section summarizes the final opinion of the committee after the discussion and is the basis for the assigned Overall Impact/Priority score.

CRITIQUE 1

Significance:	1
Investigator(s):	1
Innovation:	1
Approach:	4
Environment:	1

DESCRIPTION (provided by reviewer): This hybrid type-II proposal is entitled “Scaling Up Implementation Strategies to Improve the DIAGNOSE and PREVENT Pillars for Young MSM in Florida” and is an MPI submission by Naar, Fernández, and Bauermeister. Florida had the highest number of new HIV infections in the U.S. in 2019, and the team plans to implement this project in the 7 priority EHE jurisdictions located across Florida. This project’s primary aims are to 1) improve the quality of counseling, testing, and referral (CTR) practices of 42 agencies providing HIV testing (i.e., implementation outcome) and 2) increase rates of HIV testing among young men who have sex with men (i.e., effectiveness outcome) using the EPIS model. Building from their prior research funded by several NIH grants and the ATN, the team will implement intervention strategies to increase the skillset of the CTR workforce by training the workforce to use tailored motivational interviewing that is culturally and developmentally tailored for YMSM. Moreover, technical assistance will be provided to the agencies to identify and address inner and outer context factors that impact workforce development (e.g., workplace climate). Mystery shoppers will be used to guide and evaluate the success of intervention activities, wherein YMSM will evaluate the services received at each agency twice quarterly throughout the project (pre-, during, and post-implementation) using standardized assessments developed by the team in prior research. More specifically, the team will use a blocked step-wedge design to roll out their intervention strategy and conduct outcome assessments up to 12-months post-implementation. At 12-months post implementation, agencies will be randomized again to either continue the current intervention or move to an observation-only arm to help guide sustainability outcome evaluation (secondary Aim 1). The team will also interview up to five providers per agency immediately post-implementation and again immediately post-sustainment to determine barriers and facilitators to successful implementation of evidence-based practices aligned with inner and outer setting factors of the EPIS model. The team will identify changes in HIV testing using Florida Dept. of Health surveillance data. As surveillance data recently begun collecting PrEP uptake, the team will also assess changes in PrEP use among YMSM as an exploratory outcome; PrEP referral is an integral component of the CTR workforce training, in addition to risk reduction counseling and tailored motivational interviewing.

Overall Impact: This application was overall considered an outstanding proposal. The team is well-qualified to carry out the proposed research in CTR agencies throughout the 7 jurisdictions in Florida. This project builds off additional funded work in this area focused on treatment outcomes, and it is an extension of existing infrastructure of academic-community partnerships to address HIV prevention and diagnosis components of the EHE strategy. The investigators make a really strong case with their

preliminary data about how woefully incompetent CTR agencies are at providing motivational interviewing to YMSM—a core component of risk reduction and pre- and post-test counseling practices for HIV testing centers. Moreover, the investigators highlight just how important youth-centered mystery shoppers can be at identifying areas for clinics to improve their clinical environments to be supportive of YMSM. Given the limited attention to improving HIV counseling with current evidence-based practices using implementation science, this project is highly innovative and impactful with the strategies they plan to deploy. A score-driving weakness of this proposal was the limited amount of detail provided about how the intervention strategy will target improving clinic procedures as identified in their logic model. The investigators appear to include the scope of this work in a single catch-all statement of the technical assistance intervention component, which seems inadequate for the potential scope of this work at the structural level. Nonetheless, this is a minor weakness in the context of the novelty and impact of the attention to improving CTR practices to score this application in the high-impact range.

1. Significance:

Strengths

- The project aims to address a critical barrier to HIV testing and PrEP referral by improving the quality of HIV test counseling in real-world practice settings.
- Successful completion of the aims could have major implications for CTR workforce training and quality control evaluation using the proposed tailored motivational interviewing training and mystery shopping practices.
- The project priorities implementation by using a stepped-wedge design, which will also allow joint determination of effectiveness and implementation outcomes.

Weaknesses

- None were noted.

2. Investigators:

Strengths

- A strong team of investigators with expertise in implementation science, intervention development and deployment, evaluation, multi-site coordination, biostatistics, qualitative and mixed-methods analysis and psychology.
- Some investigators are geographically located to provide local oversight of the intervention activities within their region of the Florida.
- Investigators have a strong history of collaboration within the ATN.

Weaknesses

- None were noted.

3. Innovation:

Strengths

- There is currently little work around improving HIV test counseling using an implementation science lens.
- The use of mystery shoppers is innovative and impactful for assessing clinical and structural changes of HIV testing environments.

- This proposal is youth centric by including YMSM in evaluation activities, as well as the support an inclusion of the FLASH network as a community advisory board.
- Although motivational interviewing is no longer innovative, the implementation of tailored MI is highly innovative and needed given lackluster performance of HIV testing centers in providing competent care to YMSM.

Weaknesses

- None were noted.

4. Approach:

Strengths

- The hybrid type-II designs using a stepped wedge randomized trial is rigorous in the context of joint and equal attention to the assessment of effectiveness and implementation outcomes.
- Implementing tailored motivational interviewing is highly impactful.
- Including YMSM in evaluation activities as mystery shoppers is innovative and impactful.
- Post-implementation reassignment is impactful for determining sustainability of intervention activities.
- The project is feasible based on the scope of the investigators' prior work.
- Strong fidelity procedures integrated throughout the proposal.

Weaknesses

- There were limited details provided about how the intervention strategy will address the structural factors in the logic model (i.e., improvement of clinic procedures).
- 12-months post-implementation does not seem very long to see meaningful changes in HIV testing rates, given retesting mostly recommended every 6 months, and it is unclear what type of effect size is anticipated for bringing in new patients to these CTR sites based on "patient shopping" and social network referral.
- Tailored motivational interview training his highly intensive, which limits potential scalability without attention to cost-effectiveness or equity outcomes.

5. Environment:

Strengths

- Strong research environment for the proposed research.
- Investigators have an existing infrastructure of collaboration within the ATN.

Weaknesses

- None were noted.

CRITIQUE 2

Significance:	4
Investigator(s):	3
Innovation:	4

Approach: 5
Environment: 3

Overall Impact: This application proposes to launch a statewide effort in Florida to promote the delivery of developmentally and culturally appropriate interventions to enhance the diagnose and prevent pillars of the HIV continuum of care for young men who have sex with men (YMSM). They propose to leverage implementation strategies to deliver PrEP referrals and tailored motivational interviewing in 42 agencies in 7 EHE priority areas in Florida. Overall strengths of the application include a focus on an understudied and underserved high-risk group of young men who have sex with men in high-risk geographical areas of Florida. The focus on this population is innovative, as well as previous work which has demonstrated involvement of the community in the proposed interventions. The study is led by a strong research team who have existing relationships with key stakeholders in the Florida Department of Health, which should increase the likelihood of successful implementation. Areas of weakness primarily lie in the Approach with additional details needed for clarification on the mystery shopper approach and its role in enhancing the implementation science strategies. Primary and secondary outcomes related to HIV testing and PrEP referral/utilization are somewhat unclear since data is not provided on those processes currently in place at the target agencies, nor any agency or individual level factors which could also affect outcomes other than the target interventions. Length of time for intervention phases across blocks of randomization is not well supported by previous research. With clarification of some of the noted limitations, the study may make an overall impact on HIV testing and PrEP referral among this high-risk and understudied group.

1. Significance:

Strengths

- The application is grounded geographically in Florida, one of the states with the highest HIV diagnoses in the US, and second in the nation for the number of EHE jurisdictions. Focusing this effort on Florida Departments of Health to maximize reach across the state is important.
- The study targets interventions which specifically address the diagnose and prevent pillars of the HIV continuum of care, which are critical for the target population.
- The focus on young sexual minority men (YSMM), particularly men of color, is critical as this is a population less likely to know they are infected and a population for which targeted developmental and culturally sensitive interventions have been lacking in the empirical literature.
- Tailoring intervention delivery strategies, such as motivational interviewing, is important for cultural and developmental impact and outcomes of study interventions.

Weaknesses

- Noted limitations exist for the HealthForce, the care team historically responsible for HIV services, particularly related to culturally and developmentally sensitive interventions. It is not clear if HealthForce is a formal service delivery system, what types of health/behavioral health services are provided by HealthForce, the current state of EBPs within this organization, nor the expectations for change. Empirical support for the possible broad impact associated with this health care delivery system is limited. Rigor of prior research is not convincing.
- The focus on implementation science seems to be more associated with the study approach rather than understanding the significance of the problem.

2. Investigators:

Strengths

- MPI Naar has experience as an NIH funded investigator with research focused on HIV prevention and treatment, implementation science to scale up interventions, motivational interviewing, and developmentally and culturally tailored interventions.
- MPI Bauermeister has research expertise in HIV prevention among high-risk adolescents and multilevel interventions with sexual and gender minority youth.
- The PI team is complemented by Dr. Aryal, the study statistician, who has experience on NIH-funded projects; Dr. Balan has research expertise in mixed methods designs and analysis and will oversee motivational interviewing adaptations, data interpretation, and clinic logistics.
- Co-I Spencer is affiliated with the Florida Department of Health, which is likely a strength for implementation.

Weaknesses

- The contribution of MPI Fernandez is a little unclear between the MPI plan and the biosketch, which seems to focus more on oversight of the mystery shoppers.

3. Innovation:

Strengths

- Studies focusing on youth-centered HIV diagnoses and prevention are limited in the empirical research.
- The work is informed by the target population and proposed strategies rely on input and feedback from members of the community.

Weaknesses

- The use of the EPIS framework is not particularly innovative in this context.
- Based on previous work and citations provided, it seems that the use of tailored motivational interviewing has been used in CTR settings and is not particularly innovative.

4. Approach:

Strengths

- Preliminary studies by the research team suggest feasible outcomes related to tailored motivational interviewing related to testing, linkage, retention, and viral suppression across multiple clinic sites. These trials also included positive outcomes for training and quality management within staff groups, as well as the mystery shopping approach to understanding tailored developmental and culturally appropriate services.
- Utilization of the EPIS framework is a strength in that it allows for feedback and input from the agencies and FDOH during the exploratory and preparation phases before implementation of interventions. On-going quality management meetings are also strengths to assess the implementation process.
- Trainings provided for TMI and MS are detailed and grounded in previous work.

Weaknesses

- It is unclear from the logic model how the mystery shopping approach enhances the proposed intervention strategies or mechanisms (training and improvement of clinic procedures). It is not

clear if target agencies are aware of the mystery shopper visits, and if/how that might change usual practice.

- It is not clear that the data collected by the mystery shoppers during the exploratory phase will yield valuable data related to collaborative consideration of the health needs of the community. Data seems to be fairly subjective – asking about how they felt about the visit and their overall perspectives – rather than assessments and procedures.
- If counseling, testing, referral is the prevention package guiding the study, it is not clear why the primary client/patient effectiveness outcome is limited to HIV testing – with exploratory outcomes grounded in PrEP initiation. Additional data on factors associated with HIV testing and PrEP initiation among youth (other than the agency level interventions) would be particularly informative here. It also seems that MS change scores will be a secondary outcome, but it is not clear how that will be measured or analyzed.
- Additional clarity is needed on the proposed intent-to-treat analysis for Aim 1 since all agencies will receive all the interventions, just at different times.
- While the stepped wedge design seems appropriate for measuring intervention implementation at the agency level, the length of the intervention phases are not well described nor justified. The rationale for different levels of time of intervention across blocks is confusing.

5. Environment:

Strengths

- Through relationships with the Florida Department of Health, the research team is well positioned to carry out this statewide effort in some of the highest HIV risk and EHE priority areas of Florida.
- Proposed academic units seem to include the necessary facilities, resources, and equipment needed to complete the proposed study.

Weaknesses

- Additional details on the HealthForce health care delivery system would have been informative.

CRITIQUE 3

Significance:	2
Investigator(s):	2
Innovation:	3
Approach:	3
Environment:	2

Overall Impact: Extremely ambitious project aimed at increasing the quality of HIV prevention counseling for young sexual minority men in Florida DOH counseling and testing centers through for motivational interviewing training for center staff. The intervention will be tested by mystery shoppers who will visit the various agencies. In addition, they will measure the number of HIV tests administered to young SMM before and after the intervention. The investigators are well qualified to carry out the study and they have partnered with and have support from the Florida DOH. They have engaged a robust youth advisory board. The intervention and the study are very complex and will require significant buy-in and effort from the agencies – the investigators seem to have done a significant amount of groundwork in this area.

1. Significance:

Strengths

- The project is aimed at the highest risk group in a state with high HIV incidence.
- The integration with the Department of Health and agencies providing counseling and testing is robust and responsive to the RFA.
- The engagement of FLASH is a strength.
- The basic intervention- the motivational interview training is scalable.

Weaknesses

- Very complex and labor intensive, some of the other aspects of the intervention (the QA, the Technical Assistance) will be more difficult to scale.

2. Investigators:

Strengths

- Investigators are highly qualified and have worked together effectively.
- There is expertise and experience in implementation science.

Weaknesses

- None identified.

3. Innovation:

Strengths

- Mystery shoppers are innovative and should be impactful on agency activities.
- The study is assessing real-world outcomes across a number of agencies with a centralized intervention and plan.

Weaknesses

- Motivational interviewing in and of itself is not innovative.

4. Approach:

Strengths

- The evaluations using number of tests is a practical real-world outcome that is easily obtained, the pre-post intervention assessment makes sense. The secondary aim of PrEP initiation using the established data collected, is efficient and an important real-world outcome.
- The technical assistance seems robust.
- The evaluation plan is sound.

Weaknesses

- The project requires significant engagement from the CTRs at many levels, while the investigators seem to have engaged many of the organizations, contingencies for unwilling or under-engaged agencies or individuals are not addressed.

5. Environment:

Strengths

- The academic environment is excellent.

Weaknesses

- As above, the study relies on effort from a wide variety of agencies, some of which may not be fully engaged.

ADDITIONAL COMMENTS TO APPLICANT:
Comments:
The number of acronyms in application was quite distracting making it difficult to wade through the application to understand the science, the interventions, and the outcomes. Please remember that on a diverse panel, there will be reviewers who don't know every term and acronym.

THE FOLLOWING RESUME SECTIONS WERE PREPARED BY THE SCIENTIFIC REVIEW OFFICER TO SUMMARIZE THE OUTCOME OF DISCUSSIONS OF THE REVIEW COMMITTEE ON THE FOLLOWING ISSUES:

PROTECTION OF HUMAN SUBJECTS: ACCEPATBLE (CODE 30)

INCLUSION OF WOMEN PLAN: ACCEPATBLE (CODE G1A)

INCLUSION OF MINORITIES PLAN: ACCEPATBLE (CODE M1A)

INCLUSION OF INDIVIDUALS ACROSS THE LIFESPAN: ACCEPATBLE (CODE C3A)

VERTEBRATE ANIMAL: NOT APPLICABLE

RESOURCE SHARING PLANS

DATA SHARING PLAN: ACCEPATBLE

MODEL ORGANISMS SHARING PLAN: NOT APPLICABLE

GENOMIC DATA SHARING PLAN: NOT APPLICABLE

AUTHENTICATION OF KEY BIOLOGICAL AND/OR CHEMICAL RESOURCES: NOT APPLICABLE

SCIENTIFIC REVIEW OFFICER'S ADMINISTRATIVE NOTES: NOT APPLICABLE

BUDGETARY OVERLAP: NOT APPLICABLE

COMMITTEE BUDGET RECOMMENDATIONS:
The budget is recommended as requested in all years.

Footnotes for 1 R01 AI169980-01; PI Name: NAAR, SYLVIE

NIH has modified its policy regarding the receipt of resubmissions (amended applications).See Guide Notice NOT-OD-18-197 at https://grants.nih.gov/grants/guide/notice-files/NOT-OD-18-197.html. The impact/priority score is calculated after discussion of an application by averaging the overall scores (1-9) given by all voting reviewers on the committee and multiplying by 10. The criterion scores are submitted prior to the meeting by the individual reviewers assigned to an application, and are not discussed specifically at the review meeting or calculated into the overall impact score. Some applications also receive a percentile ranking. For details on the review process, see http://grants.nih.gov/grants/peer_review_process.htm#scoring.

MEETING ROSTER

National Institute of Allergy and Infectious Diseases Special Emphasis Panel
NATIONAL INSTITUTE OF ALLERGY AND INFECTIOUS DISEASES
Prevention Strategies to End the HIV Epidemic (R01 Clinical Trial Optional)

ZAI1 DNV-A (J3)
11/18/2021

Notice of NIH Policy to All Applicants: Meeting rosters are provided for information purposes only. Applicant investigators and institutional officials must not communicate directly with study section members about an application before or after the review. Failure to observe this policy will create a serious breach of integrity in the peer review process, and may lead to actions outlined in NOT-OD-14-073 at https://grants.nih.gov/grants/guide/notice-files/NOT-OD-14-073.html, NOT-OD-15-106 at https://grants.nih.gov/grants/guide/notice-files/NOT-OD-15-106.html, and NOT-OD-18-115 at https://grants.nih.gov/grants/guide/notice-files/NOT-OD-18-115.html, including removal of the application from immediate review.

CHAIRPERSON(S)

STATON, MICHELE, PHD
PROFESSOR
DEPARTMENT OF BEHAVIORAL SCIENCES
COLLEGE OF MEDICINE
UNIVERSITY OF KENTUCKY
LEXINGTON, KY 40536

MEMBERS

AVERY, ANN K, MD
ASSOCIATE PROFESSOR
DEPARTMENT OF INFECTIOUS DISEASES
CASE WESTERN RESERVE UNIVERSITY
CLEVELAND, OH 44106

BOYD, DONTE, PHD
ASSISTANT PROFESSOR
COLLEGE OF SOCIAL WORK
THE OHIO STATE UNIVERSITY
COLUMBUS, OH 43210

FRENCH, AUDREY, MD
PROFESSOR OF MEDICINE
DIVISION OF INFECTIOUS DISEASE
STROGER HOSPITAL OF COOK COUNTY
CHICAGO, IL 60612

JOHN, STEVEN A, MPH, PHD
ASSISTANT PROFESSOR
DEPARTMENT OF PSYCHIATRY AND BEHAVIORAL MEDICINE
CENTER OF AIDS INTERVENTION RESEARCH
MEDICAL COLLEGE OF WISCONSIN
MILWAUKEE, WI 53202

KAPADIA, FARZANA, PHD
ASSOCIATE PROFESSOR OF GLOBAL PUBLIC HEALTH & POPULATION HEALTH
DEPARTMENT OF EPIDEMIOLOGY
SCHOOL OF GLOBAL PUBLIC HEALTH
NEW YORK UNIVERSITY
NEW YORK, NY 10003

KLEIN, CHARLES HOWARD, PHD
ASSOCIATE PROFESSOR
PORTLAND STATE UNIVERSITY
DEPARTMENT OF ANTHROPOLOGY
PORTLAND, OR 97207

LELUTIU-WEINBERGER, CORINA, PHD
ASSOCIATE PROFESSOR
RUTGERS BIOMEDICAL AND HEALTH SCIENCES
SCHOOL OF NURSING
RUTGERS UNIVERSITY
NEWARK, NJ 07107

MA, GRACE X., PHD
ASSOCIATE DEAN FOR HEALTH DISPARITIES
DEPARTMENT OF URBAN AND POPULATION SCIENCES
DIRECTOR, CENTER FOR ASIAN HEALTH
LAURA H CARNEL PROFESSOR
LEWIS KATZ SCHOOL OF MEDICINE, TEMPLE UNIVERSITY
PHILADELPHIA, PA 19122

MPOFU, ELIAS, PHD
PROFESSOR OF REHABILITATION SERVICES
COLLEGE OF HEALTH AND PUBLIC SERVICE
UNIVERSITY OF NORTH TEXAS
DENTON, TX 76201

SCHNARRS, PHILLIP W, PHD
ASSOCIATE PROFESSOR
DEPARTMENT OF POPULATION HEALTH
DIVISION OF COMMUNITY ENGAGEMENT AND HEALTH EQUITY
DELL MEDICAL SCHOOL
THE UNIVERSITY OF TEXAS AT AUSTIN
SAN ANTONIO, TX 78249

SCIENTIFIC REVIEW OFFICER

VATAKIS, DIMITRIOS N., PHD
SCIENTIFIC REVIEW OFFICER
AIDS REVIEW BRANCH
SCIENTIFIC REVIEW PROGRAM
DIVISION OF EXTRAMURAL ACTIVITIES
NATIONAL INSTITUTES OF HEALTH/NIAID
ROCKVILLE, MD 20852

WICKHAM, KRISTINA S., PHD
SCIENTIFIC REVIEW OFFICER
SCIENTIFIC REVIEW PROGRAM
DIVISION OF EXTRAMURAL ACTIVITIES
NATIONAL INSTITUTES OF HEALTH/NIAID
ROCKVILLE, MD 20852

EXTRAMURAL SUPPORT ASSISTANT

CRAWFORD, KAMESHA C.
PROGRAM SPECIALIST
AIDS REVIEW BRANCH
SCIENTIFIC REVIEW PROGRAM
DIVISION OF EXTRAMURAL ACTIVITIES
NATIONAL INSTITUTES OF HEALTH/NIAID
ROCKVILLE, MD 20852

Consultants are required to absent themselves from the room during the review of any application if their presence would constitute or appear to constitute a conflict of interest.
